# Assessment of visceral pain with special reference to chronic pancreatitis

**DOI:** 10.3389/fpain.2022.1067103

**Published:** 2022-12-20

**Authors:** Louise Kuhlmann, Søren Schou Olesen, Asbjørn Mohr Drewes

**Affiliations:** ^1^ Mech-Sense, Department of Gastroenterology and Hepatology, Aalborg University Hospital, Aalborg, Denmark; ^2^ Department of Clinical Medicine, Aalborg University, Aalborg, Denmark

**Keywords:** pain, chronic pain, visceral pain, pain characterization, chronic pancreatitis, pain assessment

## Abstract

A thorough pain assessment is of utmost importance when managing pain in clinical practice as it is the foundation for defining pain in need of treatment, either interventional or pharmacological. Pain characteristics can also guide interventional strategies and help evaluate the effect of treatment. In research settings, standardized pain assessment is crucial to improve comparability across studies and facilitate meta-analysis. Due to the importance of thorough visceral pain assessment, this manuscript describes the key elements of pain evaluation focusing on chronic pancreatitis. Most studies in pain assessment have focused on somatic pain, and although chronic pain often shares characteristics between etiologies, some differences must be addressed when assessing visceral pain. Especially differences between somatic and visceral pain are apparent, where visceral pain is diffuse and difficult to localize, with referred pain aspects and often autonomic symptoms dominating the clinical picture. These aspects need to be incorporated into the pain assessment instrument. The manuscript will discuss the different ways of assessing pain, including unidimensional measurement scales, multidimensional questionnaires, and quantitative sensory testing. The advantages and challenges linked to the different methods will be evaluated.

## Introduction

Pain is a frequent symptom in the adult population, with a prevalence of up to 20% (1). It is a common cause of seeking medical advice in primary, secondary, and tertiary health sectors. Chronic pain has significant consequences for the patient's life quality, as it affects not only physical health but also psychological well-being, daily activities, and economic functioning (2–5). Besides this, chronic pain also has enormous direct and indirect, associated societal costs (6). Pain treatment is essential in optimizing patient quality of life and disease-related cost. It can minimize pain-related admission and diminish the need for disability payments by maintaining the ability to work (5, 6).

Somatic and visceral pain have many similarities; however, the differences are also considerable. The transmission of visceral pain sensation varies from somatic pain as the afferent nerves innervating viscera terminate at several spinal levels leading to diffuse pain perception. In the projection to the spinal cord alongside sympathetic fibers, cross-talk often occurs with resultant autonomic symptoms such as nausea, sweating, early satiety, and diarrhea (7). In the spinal cord, the fibers converge with somatic fibers (8). This may lead to pain referred to somatic and other visceral structures.

Visceral diseases are typically associated with severe and disabling pain. According to the International Association for the Study of Pain (IASP) classification of chronic pain, visceral pain can be either primary (previously labeled functional) or secondary (to organic diseases) (9). Although pain is a hallmark of primary visceral pain, in this chapter, we will focus on organic pain, where the diseases are better characterized and understood, with special reference to chronic pancreatitis (CP). However, the principles mentioned in this article can typically also be used in other types of visceral pain, including irritable bowel syndrome, bladder pain syndrome, and endometriosis.

CP is a progressive fibroinflammatory disease where the dominating symptom is visceral pain (10). Pain affects up to 60%–70% of patients, affecting mental health and quality of life (11). The pathophysiology of pain in CP is multifactorial and often caused by a complex interplay between factors such as pancreatic duct obstruction, inflammation, and pancreatic neuropathy (12, 13).

Patients often describe their pain as a continuous, severe, epigastric pain radiating to the back (12), but pain localization varies between individuals (14). The pain is typically fluctuating over time, some patients have pain-free intervals, and other patients have chronic pain with exacerbations (15).

In clinical studies, pain assessment in CP varies considerably. The Pancreatitis-Quantitative Sensory Testing (P-QST) consortium (16) is currently working on a meta-analysis assessing the effect of endoscopic and surgical pain treatment in CP, and preliminary results show that although pain score improvements are similar in the two groups, there are problems with comparing the treatments, as the pain assessment differs considerably between studies. As such, pain assessment varies from comprehensive pain questionnaires to simply asking the patients how they feel. As pain relief is often the primary endpoint in interventional studies of CP, the greatly varying methods for pain assessment across studies are problematic, and studies addressing different treatments can hardly be compared.

## Pain assessment tools in chronic pancreatitis

Pain treatment is a difficult and complicated task as chronic pain patients are very heterogenous due to many different origins of pain, diversity in affected pain mechanisms, many pain-associated risk factors, differences in coping strategies, and differing responses to pharmacological treatments that, again, often are associated with many side effects (17). Due to the heterogenicity of the patient group, treatment should be individualized to fit the patient's pain phenotype, depending on, for instance, pain characteristics and affected pain mechanisms. In this context, pain assessment is essential. A scoring of pain severity is used to evaluate the need for analgesic treatment; pain management strategies can be developed from thorough pain characterization; finally, pain assessment is central in evaluating treatment effects (18).

The subjective nature of pain sensation makes objective estimation of pain intensity impossible (18). Therefore, the gold standard for pain assessment is patients’ pain self-reports. The method of the patient report can vary from verbal, unidimensional measurements to written comprehensive multidimensional pain assessment. Pain has many components, including pain intensity, localization, pattern, factors provoking pain, factors exacerbating pain, pain-related symptoms, current treatment (pharmacological), previous treatments (pharmacological as well as interventional), quality of life, mental health, and risk factors for pain. The many aspects of pain underline the need for multidimensional pain assessment.

Unidimensional pain scales such as the Visual Analogue Scale and the Numerical Rating Scale are commonly used in clinic and research practice to assess pain intensity. There are, however, several challenges in using unidimensional scales for pain assessment. The scales are simple measurements, but the interpretation reflects the individual's conceptualization of pain, resulting in significant differences between reports. It has been suggested that the unidimensional scales should be converted to ratio scales to provide information on changes over time rather than a single measurement (19). As the unidimensional scales leave several aspects of pain assessment in the dark, it is likely more suited for assessing acute rather than chronic pain when used as a stand-alone measure (18).

Different recommendations on pain assessment, including the Initiative on Methods, Measurement, and Pain Assessment in Clinical Trials (IMMPACT) and Validation and Application of a patient-relevant core set of outcome domains to assess multimodal PAIN therapy (VAPAIN) recommendations, specify that several core domains in pain should be considered in clinical studies (20, 21). When complying with these recommendations, pain assessment will provide information on pain intensity, characteristics, and how pain affects different aspects of patients’ lives, including sleep, economic function, and psychological health. In visceral pain, changes in pain characteristics can be caused by new disease-related complications, where targeted treatments might exist and are therefore mandatory to assess (22). A more in-depth assessment can provide important clinical knowledge that can be used to evaluate the need for further examinations. Multidimensional scales are, therefore, useful in visceral pain. It can focus on disease-specific characteristics and evaluate further aspects of pain if developed for a specific condition. This gives a complete image of how pain affects the patients’ lives. However, a detailed multidimensional pain characterization is time-consuming, limiting the use of comprehensive pain assessment tools in research and clinical practice.

Until recently, there has been a lack of formally validated pain assessment tools developed specifically for CP. The Izbicki pain scale has commonly been used, as it is developed specifically for CP, but it still lacks the formal validation process. It was presented in a study in 1995 and has been used extensively afterward (23). The questionnaire is quite simple, evaluating pain on intensity, frequency of pain attacks, use of analgesic treatment, and inability to work. Each subpart accounts for 25% of the score, but due to the workability assessment, it is limited in its response to treatments over shorter periods. Besides the Izbicki pain scale, other non-chronic-pancreatitis-specific questionnaires, such as the brief pain inventory, have been validated and used for pain assessment. However, these questionnaires lack evaluation of pancreatic pain-specific domains such as postprandial pain and gastrointestinal manifestations (24).

Recently a comprehensive pain assessment questionnaire, the Comprehensive Pain Assessment Tool (COMPAT), has been developed specifically for CP, complying with the IMMPACT and VAPAIN recommendations (14). It is useful for a comprehensive evaluation of pancreatic pain, but due to the extensive length of 17 pages, some patients might not be able to answer the questionnaire sufficiently. Consequently, a short form of the COMPAT questionnaire, the COMPAT-SF, has been developed (25) and validated as a separate questionnaire. The COMPAT-SF scores correlate to scores from the brief pain inventory and the Izbicki pain scale. It also correlates to patient quality of life and hospitalizations due to pain in the previous year. Reliability has been evaluated both on internal consistency and in a test-retest examination. It has been proven acceptable, especially when considering chronic pain's fluctuating nature (25). Predictive validity and the power as a decision-making tool are still lacking but will be examined in future years.

An international guideline for using different pain questionnaires and recommendations for their use in painful CP has recently been published. For further details, the reader is referred to (26).

## Neurophysiological assessment of pain in chronic pancreatitis

**Figure 1:**
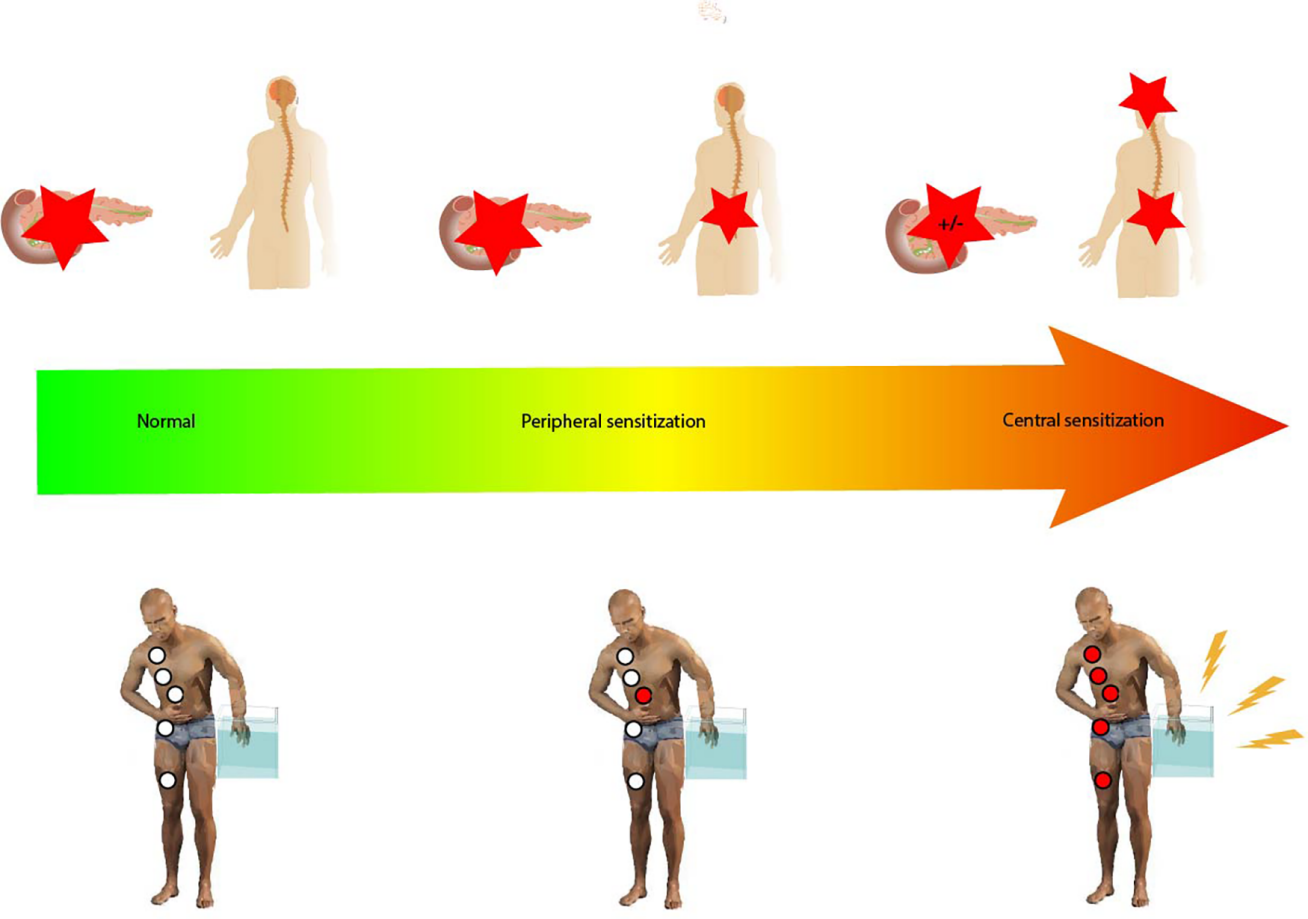
Pain responses ranging from normal through peripheral sensitization to central sensitization. Peripheral sensitization is characterized by increased excitability of the second-order neurons sharing spinal innervation with the pancreatic gland but lacking changes in the central pain processing pathways. Central sensitization is associated with changes in central pain processing pathways. The red stars indicate the generation of a nociceptive response to stimulation, either peripherally or centrally. Hypersensitivity as tested by the P-QST study protocol (pain thresholds as well as temporal summation) is indicated by the red circles; white circles indicate normal P-QST responses. In central sensitization, dynamic QST measures such as conditioned pain modulation can also be affected as shown in the figure.

Questionnaires can however fail to capture the complexity of visceral pain in CP, and research has focused on identifying additional methods for assessing pain and guiding treatment strategies (27). Quantitative Sensory Testing (QST) can be used to assess pain, where it serves to characterize sensory processing in both peripheral and central pain pathways (Figure 1). It can serve as a means to phenotype the patient's nociceptive profile. In QST, standardized stimulations of somatic and visceral tissue are used to explore different neural pathways and networks. This results in a response quantified with psychophysical and/or objective methods (28). Visceral stimulations of patients are often not well accepted in a clinical setting, and due to convergence between visceral afferents from the pancreas and somatic afferents from the T10 dermatome, QST of the skin can be used to assess whether pain processing from the pancreas to the central nervous system is sensitized (29). In addition, when adding more specific examinations, such as assessment of endogenous descending inhibition from centers in the brainstem and temporal summation, we can analyze whether pain processing in the central pathways is abnormal. QST can be used as a biomarker to categorize pain phenotypes based on affected pain mechanisms (30).

Quantitative sensory testing involves several tests to enlighten the sensory function of pain perception, from peripheral stimulation to evaluation of the processing in the brain. The tests consist of different standardized stimulations and evaluate patients’ subjective pain intensity response. The stimulation can, for instance, be thermal, mechanical, electrical, chemical, or ischemic (31). The resulting pain intensity registration can be supplied with electroencephalography (EEG) to examine an objective measure. Resting state EEG can be used to examine the brain's default mode, whereas EEG during a painful stimulation in QST gives us information on evoked brain potentials as a result of the pain perception (32). Autonomic reactivity to nociceptive input can also be measured objectively, where heart rate variability is a promising measure (33). It changes due to increased sympathetic-baroreflex activity and a decrease in vagal-parasympathetic activity.

QST, EEG, and functional magnetic resonance imaging have been used in several clinical studies and can be used to identify different dysfunctional pain mechanisms. However, pain itself is a subjective sensation and should be assessed as such (34–36). The German Research Network on Neuropathic Pain has published guidelines on comprehensive QST batteries to examine pain thoroughly (37). Although this gives a detailed description of pain processing, it is unsuitable for clinical practice and cannot be used as a bedside tool to examine visceral pain. Invasive visceral stimulations are also used to examine pain processing in visceral pain (34, 38, 39). These examinations may involve significant discomfort for the patients, fragile and expensive equipment and are therefore not optimal for bedside use in clinical routine work. However, as visceral pain processing can be partly reflected in convergent somatic dermatomes, this can be used as a proxy of central aspects of visceral pain (40). The P-QST consortium was formed to develop and promote knowledge of a bedside QST examination that could be used to evaluate pain processing (16). It consists of three simple tests. One examines sensitization in several anatomical regions using pain detection and tolerance thresholds to standardized pressure (27, 41). If the nervous structures in anatomical regions associated with the pancreas are evaluated as sensitized, and other regions have normal thresholds, it could indicate segmental hyperalgesia corresponding to the pancreatic segment of the spinal cord. If pain thresholds are affected at several sites, it indicates generalized hyperalgesia where the damage is not only located in the peripheral nerve and associated spinal segments but also changes in the central processing of painful stimulations (42).

Another test in P-QST examines the temporal summation score. First, a single pinprick stimulation is performed on a somatic structure, such as the dominant forearm, the pancreatic dermatome (T10), or both, and the patient's corresponding pain intensity is reported. Afterward, a repetitive series of pinpricks are performed one second apart, and the resulting pain intensity is reported. The ratio between the two pain intensities is calculated as the temporal summation score (43). The final P-QST examination evaluates conditioned pain modulation, the change in pain perception after a conditioning stimulus. Conditioned pain modulation has previously been shown to correlate to clinical pain intensity and is an important aspect to include in the neurophysiological examination (41). In the P-QST consortium, the cold pressor test is recommended where a conditioning stimulus, lowering the patient's hand in water with a temperature of 2 degrees for 2 min, is performed. The test stimulus, pressure stimulation on the thigh until the pain tolerance level is reached, is performed before and immediately after the conditioning stimulus and the two values are compared (28).

The P-QST consortium has proposed an algorithm for identifying central and segmental sensitization, where at least 2 out of the following 4 measures (conditioned pain modulation, cold pressor endurance time, sum of pain detection thresholds, and temporal summation on the forearm) indicate central sensitization, whereas 1 out of the following 2 measures (1: Ratio between pain thresholds in T10 and control sites and 2: Enhanced temporal summation at the upper abdominal) indicate segmental sensitization (27).

Over the last decades of pain research, it has been debated whether QST results are gender-specific, as evidence has pointed to a difference in pain perception and pain frequency between males and females (44, 45). A systematic review by Racine et al. from 2012 did however not find results proving lower pain tolerance in women, as there was significant differences in results when comparing different test stimuli and stimulation sites (46). Research does point to variations due to the menstrual cycle, and QST measures should optimally be standardized in regards to menstrual phase (47). This would however be difficult to plan in clinical practice.

In the P-QST consortium, the reference values for pressure pain detection threshold has been differentiated between sexes, as studies show variation in this exact test stimulus (27, 46).

The reliability of QST has been discussed. Static measurements, such as pressure pain stimulation, have acceptable reproducibility, whereas dynamic parameters, especially conditioned pain modulation, show variability over time (28). However, the variability differs between stimulation methods, and there are several ways to improve reliability. This includes, among other, comprehensive training of both examinators and patients and choosing the most reproducible painful stimulation; if this is considered, the assessment method is still a potent prognostic factor in clinical studies (48, 49). The test stimulus in the conditioned pain modulation regimen was chosen due to its reproducibility as a static examination. It is the most commonly used test stimulus and is well tolerated. Although reliability varies according to test site, both inter- and intrasession reliability are generally good (43, 48). The conditioning stimulus can be of different types, as well as different intensities. It can be discussed whether the stimulus can be too painful for certain individuals, and thereby possibly excluding them from completing the stimulus. On the contrary, the stimulus also can be too mild to evaluate pain modulation. Generally, there is low reproducibility for dynamic QST (the conditioning stimulus) due to the complex mechanisms of pain modulation, and the results must be evaluated with this in mind (43). It is however accessible and easy to control, and although some patients might not endure the full conditioning stimulation, this is also a usable result in the final evaluation, as described above.

In recent studies, QST has been used to predict the outcomes of treatments. Olesen et al. have shown that hyperalgesia to electrical stimulations in the T10 dermatome is predictive of the efficacy of pregabalin treatment in CP (50). QST has also been used as a predictor in other types of patients, including diabetic neuropathy, where conditioned pain modulation predicts the efficacy of duloxetine (51), and a mixed group of chronic pain patients, where cold pain intensity and EEG activity induced by cold pain, predicted the pain reduction of opioid treatment (52). Further studies are, however, needed before using this as a decision-making tool in clinical practice, although different pain treatment algorithms using neurophysiological evaluations have been proposed (29, 53).

## Conclusion

Pain assessment in visceral pain is complex. As pain affects life in many ways, several aspects besides pain intensity must be evaluated. These include mental health, autonomic symptoms, and quality of life. Unidimensional scales are mostly suited for evaluating changes in pain intensity in acute pain but are too simple for assessing the complexity of chronic visceral pain. Questionnaire validity is increased when developed for a specific disease, as it can provide information beyond general characteristics.

Besides pain questionnaires, QST is gaining ground. It is used to quantify the loss or gain of sensory function and can be performed as a quick bedside examination with only a few instruments available. It can help evaluate the progression of chronic pain from a segmental to a central origin. In the future, it might also help tailor analgesic treatment focusing on affected pain mechanisms (29).

It's important to strive for a uniform assessment of pain in clinical studies, as this will increase the comparability of results. For further information on pain assessment in CP, please see the international guidelines on the subject (26).

## References

[1] Reid KJ Harker J Bala MM Truyers C Kellen E Bekkering GE Epidemiology of chronic non-cancer pain in Europe: narrative review of prevalence, pain treatments and pain impact. Curr Med Res Opin. (2011) 27(2):449–62. 10.1185/03007995.2010.545813 21194394

[2] Magni G Marchetti M Moreschi C Merskey H Luchini SR. Chronic musculoskeletal pain and depressive symptoms in the national health and nutrition examination I. Epidemiologic follow-up study. Pain. (1992) 53:163–8. 10.1016/0304-3959(93)90076-2 8336986

[3] Viane I Crombez G Eccleston C Devulder J De Corte W. Acceptance of the unpleasant reality of chronic pain: effects upon attention to pain and engagement with daily activities. Pain. (2004) 112(3):282–8. 10.1016/j.pain.2004.09.008 15561383

[4] Teasell RW Bombardier C. Employment-related factors in chronic pain and chronic pain disability. Clin J Pain. (2001) 17(4 Suppl):839–45. 10.1097/00002508-200112001-00010 11783830

[5] Bonathan C Hearn L Williams AC. Socioeconomic status and the course and consequences of chronic pain. Pain Manag. (2013) 3(3):159–62. 10.2217/pmt.13.18 24654756

[6] Turk DC Wilson HD Cahana A. Treatment of chronic non-cancer pain. Lancet. (2011) 377(9784):2226–35. 10.1016/S0140-6736(11)60402-9 21704872

[7] Drewes AM Olesen AE Farmer AD Szigethy E Rebours V Olesen SS. Gastrointestinal pain. Nat Rev Dis Prim. (2020) 6(1):1–16. 10.1038/s41572-019-0135-7 31907359

[8] Gebhart GF Bielefeldt K. Physiology of visceral pain. Compr Physiol. (2016) 6(4):1609–33. 10.1002/cphy.c150049 27783853

[9] Aziz Q Giamberardino MA Barke A Korwisi B Baranowski AP Wesselmann U The IASP classification of chronic pain for ICD-11: chronic secondary visceral pain. Pain. (2019) 160(1):69–76. 10.1097/j.pain.0000000000001362 30586073

[10] Majumder S Chari ST. Chronic pancreatitis. Lancet. (2016) 387(10031):1957–66. 10.1016/S0140-6736(16)00097-0 26948434

[11] Olesen SS Poulsen JL Drewes AM Frøkjær JB Laukkarinen J Parhiala M The scandinavian baltic pancreatic club (SBPC) database: design, rationale and characterisation of the study cohort. Scand J Gastroenterol. (2017) 52(8):909–15. 10.1080/00365521.2017.1322138 28471312

[12] Poulsen JL Olesen SS Malver LP Frøkjær JB Drewes AM. Pain and chronic pancreatitis: a complex interplay of multiple mechanisms. World J Gastroenterol. (2013) 19(42):7282–91. 10.3748/wjg.v19.i42.7282 24259959 PMC3831210

[13] Drewes AM Krarup AL Detlefsen S Malmstrøm M-L Dimcevski G Funch-Jensen P. Pain in chronic pancreatitis: the role of neuropathic pain mechanisms. Gut. (2008) 57(11):1616–27. 10.1136/gut.2007.146621 18566105

[14] Teo K Johnson MH Drewes AM Windsor JA. A comprehensive pain assessment tool (COMPAT) for chronic pancreatitis: development, face validation and pilot evaluation. Pancreatology. (2017) 17(5):706–19. 10.1016/j.pan.2017.07.004 28733149

[15] Mullady DK Yadav D Amann ST O’Connell MR Barmada MM Elta GH Type of pain, pain-associated complications, quality of life, disability and resource utilisation in chronic pancreatitis: a prospective cohort study. Gut. (2011) 60(1):77–84. 10.1136/gut.2010.213835 21148579 PMC6748627

[16] Phillips AE Faghih M Singh VK Olesen SS Kuhlmann L Novovic S Rationale for and development of the pancreatic quantitative sensory testing consortium to study pain in chronic pancreatitis. Pancreas. (2021) 50(9):1298–304. 10.1097/MPA.0000000000001912 34860815

[17] Breivik H Collett B Ventafridda V Cohen R Gallacher D. Survey of chronic pain in Europe: impact on daily life, and treatment. Eur J Pain. (2006) 10:287–333. 10.1016/j.ejpain.2005.06.009 16095934

[18] Breivik H Borchgrevink PC Allen SM Rosseland LA Romundstad L Breivik Hals EK Assessment of pain. Br J Anaesth. (2008) 101:17–24. 10.1093/bja/aen103 18487245

[19] Hartrick CT Kovan JP Shapiro S. The numeric rating scale for clinical pain measurement: a ratio measure? Pain Pract. (2003) 3(4):310–6. 10.1111/j.1530-7085.2003.03034.x 17166126

[20] Turk DC Dworkin RH Allen RR Bellamy N Brandenburg N Carr DB Core outcome domains for chronic pain clinical trials: iMMPACT recommendations. Pain. (2003) 106(3):337–45. 10.1016/j.pain.2003.08.001 14659516

[21] Kaiser U Kopkow C Deckert S Neustadt K Jacobi L Cameron P Developing a core outcome domain set to assessing effectiveness of interdisciplinary multimodal pain therapy: the VAPAIN consensus statement on core outcome domains. Pain. (2018) 159(4):673–83. 10.1097/j.pain.0000000000001129 29300277

[22] D’Haese JG Ceyhan GO Demir IE Tieftrunk E Friess H. Treatment options in painful chronic pancreatitis: a systematic review. HPB. (2014) 16(6):512–21. 10.1111/hpb.12173 24033614 PMC4048072

[23] Bloechle C Izbicki JR Knoefel WT Kuechler T Broelsch CE. Quality of life in chronic pancreatitis, results after duodenum-preserving resection of the head of the pancreas. Pancreas. (1995) 11(1):77–85. 10.1097/00006676-199507000-00008 7667246

[24] Tan G Jensen MP Thornby JI Shanti BF. Validation of the brief pain inventory for chronic nonmalignant pain. J Pain. (2004) 5(2):133–7. 10.1016/j.jpain.2003.12.005 15042521

[25] Kuhlmann L Teo K Olesen SS Phillips AE Faghih M Tuck N Development of the comprehensive pain assessment tool short form for chronic pancreatitis: validity and reliability testing. Clin Gastroenterol Hepatol. (2022) 20(4):e770–83. 10.1016/j.cgh.2021.05.055 34089847

[26] Drewes AM Bellin MD Besselink MG Bouwense SA Olesen SS van Santvoort H Assessment of pain associated with chronic pancreatitis: an international consensus guideline. Pancreatology. (2021) 21(7):1256–84. 10.1016/j.pan.2021.07.004 34391675

[27] Phillips AE Faghih M Kuhlmann L Larsen IM Drewes AM Singh VK A clinically feasible method for the assessment and characterization of pain in patients with chronic pancreatitis. Pancreatology. (2020) 20(1):25–34. 10.1016/j.pan.2019.11.007 31787527

[28] Olesen SS van Goor H Bouwense SAW Wilder-Smith OHG Drewes AM. Reliability of static and dynamic quantitative sensory testing in patients with painful chronic pancreatitis. Reg Anesth Pain Med. (2012) 37(5):530–6. 10.1097/AAP.0b013e3182632c40 22854397

[29] Kuhlmann L Olesen SS Olesen AE Arendt-Nielsen L Drewes AM. Mechanism-based pain management in chronic pancreatitis – is it time for a paradigm shift? Expert Rev Clin Pharmacol. (2019) 12(3):1–10. 10.1080/17512433.2019.1571409 30664364

[30] Smith SM Dworkin RH Turk DC Baron R Polydefkis M Tracey I The potential role of sensory testing, skin biopsy, and functional brain imaging as biomarkers in chronic pain clinical trials: iMMPACT considerations. J Pain. (2017) 18(7):757–77. 10.1016/j.jpain.2017.02.429 28254585 PMC5484729

[31] Cruz-Almeida Y Fillingim R. Can quantitative sensory testing move us closer to mechanism-based pain management. Pain Med. (2012) 100(2):130–4. 10.1111/pme.12230 PMC3947088 24010588

[32] Bouwense SAW de Vries M Schreuder LTW Olesen SS Frøkjær JB Drewes AM Systematic mechanism-orientated approach to chronic pancreatitis pain. World J Gastroenterol. (2015) 21(1):47–59. 10.3748/wjg.v21.i1.47 25574079 PMC4284360

[33] Koenig J Jarczok MN Ellis RJ Hillecke TK Thayer JF. Heart rate variability and experimentally induced pain in healthy adults: a systematic review. Eur J Pain. (2014) 18(3):301–14. 10.1002/j.1532-2149.2013.00379.x 23922336

[34] Arendt-Nielsen L Yarnitsky D. Experimental and clinical applications of quantitative sensory testing applied to skin, muscles and viscera. J Pain. (2009) 10(6):556–72. 10.1016/j.jpain.2009.02.002 19380256

[35] Olesen SS Hansen TM Graversen C Steimle K Wilder-Smith OHG Drewes AM. Slowed EEG rhythmicity in patients with chronic pancreatitis: evidence of abnormal cerebral pain processing? Eur J Gastroenterol Hepatol. (2011) 23(5):418–24. 10.1097/MEG.0b013e3283457b09 21399506

[36] Olesen SS Hansen TM Graversen C Valeriani M Drewes AM. Cerebral excitability is abnormal in patients with painful chronic pancreatitis. Eur J Pain. (2013) 17(1):46–54. 10.1002/j.1532-2149.2012.00155.x 22508470

[37] Maier C Baron R Tölle TR Binder A Birbaumer N Birklein F Quantitative sensory testing in the German research network on neuropathic pain (DFNS): somatosensory abnormalities in 1236 patients with different neuropathic pain syndromes. Pain. (2010) 150(3):439–50. 10.1016/j.pain.2010.05.002 20627413

[38] Dimcevski G Staahl C Andersen SD Thorsgaard N Funch-Jensen P Arendt-Nielsen L Assessment of experimental pain from skin, muscle, and esophagus in patients with chronic pancreatitis. Pancreas. (2007) 35(1):22–9. 10.1097/mpa.0b013e31805c1762 17575541

[39] Dimcevski G Schipper KP Tage-Jensen U Funch-Jensen P Krarup AL Toft E Hypoalgesia to experimental visceral and somatic stimulation in painful chronic pancreatitis. Eur J Gastroenterol Hepatol. (2006) 18(7):755–64. 10.1097/01.meg.0000223903.70492.c5 16772833

[40] Arendt-Nielsen L Laursen RJ Drewes AM. Referred pain as an indicator for neural plasticity. Prog Brain Res. (2000) 129:343–56. 10.1016/S0079-6123(00)29026-2 11098702

[41] Kuhlmann L Olesen SS Grønlund D Olesen AE Phillips AE Faghih M Patient and disease characteristics associate with sensory testing results in chronic pancreatitis. Clin J Pain. (2019) 35(9):786–93. 10.1097/AJP.0000000000000740 31268890 PMC6693925

[42] Arendt-Nielsen L Morlion B Perrot S Dahan A Dickenson A Kress HGG Assessment and manifestation of central sensitisation across different chronic pain conditions. Eur J Pain. (2018) 22(2):216–41. 10.1002/ejp.1140 29105941

[43] Marcuzzi A Wrigley PJ Dean CM Adams R Hush JM. The long-term reliability of static and dynamic quantitative sensory testing in healthy individuals. Pain. (2017) 158(7):1217–23. 10.1097/j.pain.0000000000000901 28328574

[44] Kindler LL Valencia C Fillingim RB George SZ. Sex differences in experimental and clinical pain sensitivity for patients with shoulder pain. Eur J Pain. (2011) 15(2):118–23. 10.1016/j.ejpain.2010.06.001. 20598598 PMC2965801

[45] Bulls HW Freeman EL Anderson AJ Robbins MT Ness TJ Goodin BR. Sex differences in experimental measures of pain sensitivity and endogenous pain inhibition. J Pain Res. (2015) 8:311–20. 10.2147/jpr.s84607 26170713 PMC4494610

[46] Racine M Tousignant-Laflamme Y Kloda LA Dion D Dupuis G Choinière M. A systematic literature review of 10 years of research on sex/gender and experimental pain perception-part 1: are there really differences between women and men? Pain. (2012) 153:602–18. 10.1016/j.pain.2011.11.025 22192712

[47] Riley JL III Robinson ME Wise EA Price DD. A meta-analytic review of pain perception across the menstrual cycle. Pain. (1999) 81:225–35. 10.1016/S0304-3959(98)00258-9 10431710

[48] Kennedy DL Kemp HI Ridout D Yarnitsky D Rice ASC. Reliability of conditioned pain modulation: a systematic review. Pain. (2016) 157(11):2410–9. 10.1097/j.pain.0000000000000689 27559835 PMC5228613

[49] O’Neill S O’Neill L. Improving QST reliability - more raters, tests, or occasions? A multivariate generalizability study. J Pain. (2015) 16(5):454–62. 10.1016/j.jpain.2015.01.476 25683899

[50] Olesen SS Graversen C Bouwense SAW van Goor H Wilder-Smith OHG Drewes AM. Quantitative sensory testing predicts pregabalin efficacy in painful chronic pancreatitis. PLoS One. (2013) 8(3):e57963. 10.1371/journal.pone.0057963 23469256 PMC3585877

[51] Yarnitsky D Granot M Nahman-Averbuch H Khamaisi M Granovsky Y. Conditioned pain modulation predicts duloxetine efficacy in painful diabetic neuropathy. Pain. (2012) 153(6):1193–8. 10.1016/j.pain.2012.02.021 22480803

[52] Grosen K Olesen AE Gram M Jonsson T Kamp-Jensen M Andresen T Predictors of opioid efficacy in patients with chronic pain: a prospective multicenter observational cohort study. PLoS One. (2017) 12(2):1–13. 10.1371/journal.pone.0171723 PMC5291530 28158269

[53] Drewes AM Bouwense SAW Campbell CM Ceyhan GO Delhaye M Demir IE Guidelines for the understanding and management of pain in chronic pancreatitis. Pancreatology. (2017) 17(5):720–31. 10.1016/j.pan.2017.07.006 28734722

